# Oral labetalol versus oral nifedipine for the management of postpartum hypertension a randomized control trial

**DOI:** 10.12669/pjms.35.5.812

**Published:** 2019

**Authors:** Jahanara Ainuddin, Fariha Javed, Sarah Kazi

**Affiliations:** 1 Prof. Dr. Jahanara Ainuddin FCPS, Ph.D. Department of Obstetrics and Gynecology, Dow University of Health Sciences, Karachi, Pakistan; 2 Dr. Fariha Javed, MBBS. Postgraduate Resident, Department of Obstetrics and Gynecology, Dow University of Health Sciences, Karachi, Pakistan; 3 Dr. Sarah Kazi, MRCOG, FCPS. Associate Professor, Department of Obstetrics and Gynecology, Dow University of Health Sciences, Karachi, Pakistan

**Keywords:** Labetalol, Nifedipine, Postpartum hypertension

## Abstract

**Objective:**

To compare the efficacy of oral Labetalol versus oral Nifedipine for the treatment of postpartum hypertension.

**Methods:**

A prospective randomized controlled trial with parallel assignment was conducted in the department of Obstetrics and Gynecology, Dow University of Health Sciences Karachi, Pakistan, 124 patients with post partum hypertension were selected and randomized into two groups with 62 patients receiving Labetalol and 62 receiving long acting nifedipine. Initial blood pressures were recorded, and the respective drug was administered. Dose adjustments were performed in the initial 24 hours. The outcome was measured in the form of drug efficacy by lowering of systolic blood pressure less than 140mm of Hg and diastolic less than 90mm of Hg up to 48 hours after starting treatment. Data was entered and analyzed through SPSS version 20.

**Results:**

Our study randomized 62 women to oral labetalol and 62 women to oral long acting nifedipine. The time required to achieve blood pressure control was 35.6±2.8 hours in labetalol group and 30.4±1.9 hours in nifedipine group (p=0.04).length of hospital stay, need of additional antihypertensive medications were same in both groups. Minor side effects were observed more in nifedipine group.

**Conclusion:**

We conclude that both oral labetalol and oral long acting nifedipine are effective and well tolerated interventions for the management of post-partum hypertension. However we found Nifidipine more effective in the management of postpartum hypertension.

## INTRODUCTION

Hypertension complicates 6 – 10% of all Pregnancies and is a major cause of maternal morbidity and mortality.^1^ Blood pressure(BP) levels remain low immediately post delivery but gradually rise, reaching a peak three to six days after delivery. Hypertensive disorders of pregnancy may persist post-partum or appear for the first time after delivery.^2^,^3^ The national institute of health and care excellence (NICE) and American college of Obstetricians and Gynecologists (ACOG) propose treatment of post-partum hypertension when BP levels are ≥150/100mmHg checked on two consecutive occasions 4 to 6 hours apart.^4^,^5^ Failure to treat severe hypertension in the post-partum period may cause eclampsia and fatal intracranial hemorrhage.^6^ Therefore effective and urgent treatment of post-partum hypertension is recommended. A Cochrane review done in January, 2013 showed a paucity of available clinical evidence in terms of randomized controlled trials comparing treatment options for postpartum hypertension.^7^

Available pharmacological interventions for treatment of post-partum hypertension include intravenous hydralazine, intravenous or oral labetalol, nifedipine as short acting and extended long acting preparations, amlodipine and ACE inhibitors. NICE recommends that due to side effects of associated sedation, postural hypotension and depression methyldopa should be switched to an alternative agent in the post-partum period, although it was considered as the preferred drug in breast feeding mothers.^8^ Hydralazine is associated with more side effects is better not used as first line therapy to control hypertension in pregnancy and the post-partum period. The ACOG committee (February 2019) recommends using immediate release oral nifedipine as first line therapy when intravenous access is not available or obtained for treatment of severe intra-partum or post-partum hypertension. An alternative option was oral labetalol for management of severe hypertension.^9^ Previous studies have compared intravenous labetalol, hydralazine, methyldopa and immediate release oral nifedipine for the management of post-partum hypertension.^10^-^13^

In our limited resourced healthcare setting we see a large number of women with hypertension during pregnancy or in the postpartum period. In these patient’s oral labetalol and oral extended release or long acting nifedipine would be the most convenient medications for management of post-partum hypertension. The aim of our study was to evaluate the efficacy of oral labetalol versus oral nifedipine for the management of post-partum hypertension.

## METHODS

A prospective randomized controlled trial with parallel assignment was conducted in the Department of Obstetrics and Gynecology, Dow University of Health Sciences Karachi, Pakistan, between January, 2015 and December, 2015. The trial was registered at clinical trial.gov. registration no. NCT02426177.

All women with any parity and age, who delivered at ≥20 weeks of pregnancy with persistent post-partum hypertension defined as systolic blood pressure (SBP) blood pressure of ≥ 150mmHg and/or diastolic blood pressure (DBP) of ≥100mmHg requiring an antihypertensive medication were included in the study. Eligible patients had persistent hypertension following gestational hypertension, pre-eclampsia, or had denovo post-partum hypertension without previous history. Exclusion criteria were women with a history of heart block or arrhythmias, heart failure, asthma, uncontrolled diabetes, hypothyroidism, chronic hypertension with history of other pre-pregnancy antihypertensive medication intake, renal disease with Serum Creatinine level >1mg/dl, those with allergies to either nifedipine or labetalol and those not willing to participate in the study.

Eligible candidates provided written informed consent at enrollment and the study protocol was approved by the Dow University of Health Sciences Institutional Review Board. (Ref. No. IRB-S41/DUHS/-14).

Participants of the study were randomized into two groups (1) labetalol and (2) long acting nifedipine. The study was not blinded due to different types of medications and their dosage schedule. However, the selection of drug was blinded as the patients enter the study after randomization. Envelopes with drugs were numbered consecutively and sealed and jumbled up, half containing labetalol and the other half containing nifedipine. The envelops were used in patients as they entered the study after simple randomization. The Patients initial blood pressure was recorded using a mercurial sphygmomanometer and the drug assigned to that patient was given. Serial blood pressure measurements were done hourly for six hours and then four hourly till 24 hours. Labetalol was initiated 100mg per orally Q1D and increased to 1200mg as needed to control blood pressure. Nifedipine long acting (nifedipine LA 30mg) was given 30 mg per orally once daily and increased to 90mg daily as needed to control the blood pressure. If blood pressure was not controlled with the maximum dose of one medication, the other medication was added at the lowest starting dose and incremental doses given accordingly till the desired blood pressure was achieved. Desired blood pressure taken as adequate control was systolic blood pressure below 150mmHg and diastolic blood pressure between 80 and 100mmHg. Additional medications as intravenous antihypertensive and magnesium sulphate for seizure prophylaxis were used as required on an individual patient according to decision of managing team.

The primary outcome measure was drug efficacy in terms of time required to achieve blood pressure control (SBP < 150mmHg and DBO between 80 – 100mmHg), and sustained blood pressure control defined as absence of spikes of severe hypertension (SBP≤160mmHg &/or DBP ≤ 110mmHg) for at least 72 hours. Secondary outcome measures include the need of additional antihypertensive medications, length of hospital stay and drug related side effects.

Baseline demographic data including age, parity, BMI family history of hypertension, mode of delivery were collected and recorded for all participants.

Using open Epi method, the sample size was calculated with the efficacy of labetalol and nifedipine set to 81% and 58% respectively.^14^ A confidence interval of 95% with 1-beta error of 80% was set and sample size of study is N=124 was determined with 62 individuals to each group. A non-probability consecutive sampling technique was used. A dropout rate of 10% was anticipated with the target size of n=144.

Intention to treat analysis was performed and t-tests or analysis of variance were used for continuous variables and chi-square tests for categorical variables. Data was analyzed using IBM SPSS Version 20. P<0.05 was considered statistically significant.

## RESULTS

A total of 124 women were included in our study. Sixty-two women were randomized to the oral labetalol group and 62 to the oral long acting nifedipine group. The women ranged from 18 - 48 years of age. Baseline maternal characteristics were similar in both groups in terms of age, parity, body mass index and family history of hypertension. Vaginal delivery and cesarean section rates were also similar in two groups. (Table-I).

**Table I:** Demographic & Pregnancy Characteristics.

Characteristics	Labetalol	Nifedipine	P-Value

	N = 62 Mean ± SD	N = 62 Mean ± SD	
Age (Years)	26.7 ± 1.9	25.9 ± 1.8	1
Parity	3.1 ± 0.3	3.7 ± 0.7	0.57
BMI (kg/m^2^)	25.7 ± 4.05	25.5 ± 3.9	0.91

	*%*	*%*	

Family history of hypertension	22 (35%)	24 (39%)	0.97
Mode of Delivery			
Vaginal Delivery	38 (61%)	36 (58%)	1
Cesarean Section	24 (39%)	26 (42%)	0.98

**Fig.1:**
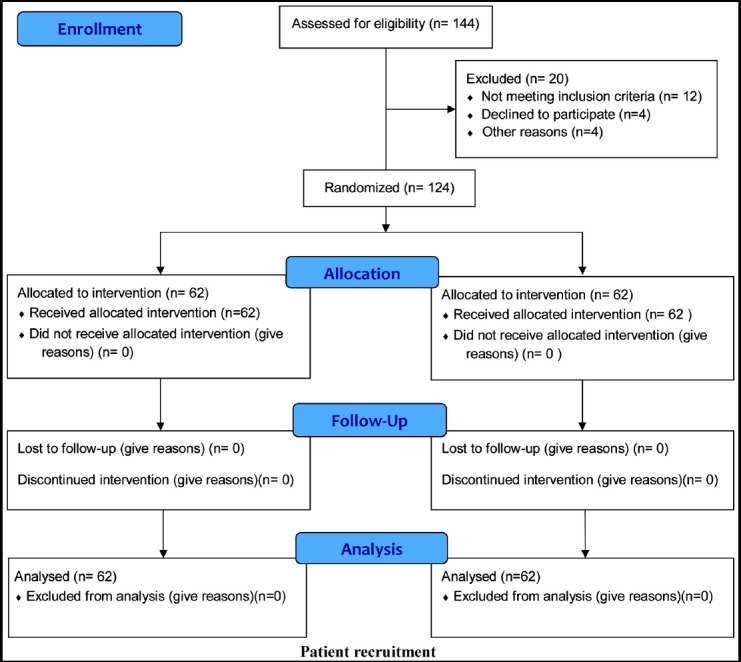
Flow Diagram.

Women in both groups completed the study. The time required to achieve blood pressure control was 35.6 ± 2.8 hours in the labetalol groups and 30.4 ± 1.9 hours in nifedipine group (p=0.041) indicating nifedipine achieved control earlier that labetalol. Similarly, blood pressure control was maintained more effectively with initial starting dose for next 72 hours in the nifedipine group compared to the labetalol group. Labetalol group required more dose increments from 100mg QDS starting dose compared to the 30mg OD nifedipine starting dose. (p=0.001) (Table-II). The need of additional oral and /or intravenous antihypertensive interventions in both groups were similar (Table-III).

**Table II:** Primary outcome (efficacy) of labetalol versus nifedipine for post-partum hypertension.

	Labetalol (n = 62)	Nifedipine (n = 62)	P-Value
1. Time Required (hours) to achieve blood pressure control (≤150/100mmHg)	35.6 ± 2.8	30.4 ± 1.9	0.041
2. Sustained blood pressure control for 72 hours	38 (61%)	54 (87%)	0.001

Reported as mean (SD), n (%).

**Table III:** Secondary outcomes of labetalol versus nifedipine for post-partum hypertension.

	Labetalol (n = 62)	Nifedipine (n = 62)	P-Value
1. Additional oral antihypertensive drug required for control	4 (6.4%)	3 (4.8%)	0.74
2. Additional intravenous antihypertensive drugs required for acute control	12 (19.4%)	10 (16.17%)	0.81
3. Length of hospital stay (days)	3.5 ± 1.3	3.4 ± 1.5	0.98

Reported as mean (SD), n (%)

In the labetalol Group-4 patients required the addition of nifedipine to control blood pressure and in the nifedipine group, three patients required the addition of labetalol once maximum drug dose was given. 58 women in labetalol group and 59 in the nifedipine group were discharged on the single agent treatment regimen initially started. The mean hospital stay was 3.5 ± 1.3 days for the labetalol group and 3.4 ± 1.5 days for the nifedipine group. Twelve women in labetalol group and 10 in in nifedipine group required one or two doses of intravenous antihypertensive drug for initial control of severe hypertension ≥ 160 / 110 mmHg. (Table-III), but later controlled on oral antihypertensive drug of the respective group. Only one woman in labetalol group required admission on 5^th^ postnatal day for uncontrolled hypertension and received an ACE inhibitor for blood pressure control.

No major side effects were observed in either group. One woman in labetalol group had bronchospasm which was managed adequately. Headache, hypotension and palpitations were common side effects noted with nifedipine. (Table-IV).

**Table IV:** Side effects of drugs.

	Labetalol (n = 62)	Nifedipine (n = 62)	P-Value
1. Bronchospasm	1 (1.6%)	0	0.005
2. Dyspnea	2 (3.2%)	2 (3.2%)	1
3. Hypotension B.P < 90/60mmHg	1 (1.6%)	5 (5.0%)	0.04
4. Palpitations	1 (1.6%)	6 (9.63%)	0.02
5. Flushing	1 (1.6%)	1 (1.6%)	1
6. Headache	4 (6.4%)	8 (12.9%)	0.05
7. Diarrhea	1 (1.6%)	0 (0%)	0.005
8. Constipation	2 (3.2%)	0	0.02

## DISCUSSION

Our study compared oral labetalol and oral long acting nifedipine for the treatment of post-partum hypertension. We found that both interventions are effective in achieving good blood pressure control with the starting dose. However, oral nifedipine achieved control earlier than oral labetalol in our study. Side effects were found to be lower in women who were given labetalol than those given nifedipine. Both drugs have minimal excretion in breast milk.^15^ Labetalol should be avoided in women with asthma as it worsens bronchospasm. A study conducted in Pakistan suggests that prevalence of asthma and related problems are high, so beta- blockers should be used with caution in our population.^16^,^17^ Similarly there are concerns regarding nifedipine related cardiovascular morbidity and mortality outside pregnancy and neuromuscular blockade with adjuvant use of magnesium sulphate in pregnancy but these were reported more with short acting nifedipine and were reported at less than 1% in controlled RCTS.^17^,^18^ The chochrane review on drugs for treatment of hypertension in pregnancy also concluded that the choice of antihypertensive agents should depend on clinician experience, discretion and suitability of patient with known adverse effects until a better evidence becomes available.^18^

Previously conducted trials compared the efficacy and safety of oral nifedipine and intravenous labetalol, hydralazine and methyldopa and found these medications effective.^18^,^19^ However there is no adequate evidence to support a particular step wise approach in the management of hypertensive disorders of pregnancy in post-partum period especially in low resource and busy settings, when long term control is desired and patient is sent back to community.^19^ Oral hypertensive agents like long acting nifedipine and labetalol are beneficial and efficacious for the long term out patient management of post-partum hypertension till adequate blood pressure control is achieved. Raheem A et al.,^10^ Vermillion et al.^20^ and Veena et al.^21^ compared the use of oral nifedipine to intravenous labetalol for managing hypertension in pregnancy and found it superior. However, there is a paucity of data comparing both oral agents. Our study demonstrates that nifedipine achieved significantly earlier and sustained post-partum blood pressure control than oral labetalol in women with elevated blood pressure. Interestingly, Sharma et al. found oral labetalol to be more effective but showed no significant difference in blood pressure control in both groups.^22^,^23^

Both studies reported no major side effects related to either drug.^22^,^23^ Previous studies also reported hypotensive episodes, palpitation and headache as common side effects associated with nifedipine use (compared to labetalol use) and these observations were noted in our study.^19^-^23^

The need for additional intravenous antihypertensive intervention for acute blood pressure control was nearly the same in both groups, as also noted in the study by Sharma et al.^23^ Length of stay and discharge time was also similar in both groups.^22^,^23^ However, Bealty et al. reported shorter hospital stay in patients treated with oral nifedipine for post-partum hypertension than those given oral labetolol.^24^

### Limitation of our study

A limitation of our study was the small number of patients and a difficulty in arranging post-discharge follow up. As a result, we cannot draw any conclusions regarding long-term management of women on either oral labetalol or nifedipine for post-partum hypertension. However, we present the first study where oral agents labetalol and nifedipine are used for the management of post-partum hypertension in a developing, resource-poor country. In addition, both patients and researchers were blinded to medication selection.

## CONCLUSION

We conclude that both oral labetalol and oral long acting nifedipine are effective and well tolerated interventions for the management of post-partum hypertension. However, nifedipine may be slightly superior due to earlier blood pressure control achieved and easier administration with once daily oral use.

### Recommendation:

Oral agents are particularly appropriate in the out-patient setting of resource constrained institutions with busy delivery suites of public hospitals in developing countries. Hypertensive disorders of pregnancy and related complications can be a major cause of maternal morbidity and mortality in these settings. The use of oral labetalol and nifedipine for the management of post-partum hypertension reduces the risk of hypertensive complications and the burden of maternal morbidity and mortality.

### Author’s Contributions

**JAA:** Designed the study, Data Analysis, Manuscript Writing.

**FJ:** Literature search, Data collection, Data Analysis.

**SK:** Data Collection, Manuscript Writing.

## References

[1] Bramhan K, Piercy N, Brown JM, Chappell LC (2013). Post-partum management of hypertension. BMJ.

[2] Podymow T, August P (2010). Post-partum course of gestational hypertension and pre-eclampsia. Hypertens Pregnancy.

[3] Goel A, Maski MR, Bajrachrya S, Wenger JB, Zhang D, Salahuddin S (2015). Epidemiology and mechanism of De NOVO and persistent hypertension in the post-partum period. Circulation.

[4] National Institute of Health Care Excellence NICE. Clinical guidelines 107:Hypertension in pregnancy:The management of hypertensive disorders during pregnancy (2011). https://www.nice.org.uk/guidance/ng133.

[5] ACOG Task force on hypertension in pregnancy. Hypertension in pregnancy (2013). Am Coll Obstet Gynecol.

[6] Lewis G, The confidential Enquiry into maternal and child Health (CEMACH) (2017). Saving mothers Lives, reviewing Maternal Deaths to make Motherhood safer 2003-2005. The seventh report on confidential requires into maternal deaths in United Kingdom London: CEMACH.

[7] Mage L, Von Dadleszen P (2013). Prevention and treatment of Post-partum hypertension. Cochrane Database Syst Rev.

[8] (2009). Medicines and Health Care products regulatory agency ACE inhibitors and angiotensin II receptor antagonists: recommendations on use during breast feeding.

[9] ACOG committee opinion, 767, Emergent therapy for acute onset, severe hypertension during pregnancy and the post-partum period (2019). Obstet Gynecol.

[10] Raheem IA, Saaid R, Omar SZ, Tan PC (2012). Oral nifedipine versus intravenous labetolol for acute blood pressure control in hypertensive emergencies of pregnancy:a randomized trial. BJOG.

[11] Griffs KR, Martin JN, Palmer SM, Martin RW, Morrison JC (1989). Utilization of hydralazine of alpha-methyldopa for the management of early puerperal hypertension. Am J Perinatal.

[12] Gracia PVD, Ruiz E, Lopez JC, de Jaramillo IA, Vega-Maleck JC, Pinzon J (2007). Management of severe hypertension in the post-partum with intravenous hydralazine or labetalol;a randomized clinical trial. Hypertens Pregnancy.

[13] Vermillion ST, Scardo JA, Naoman RB, Chauhan SP (1999). A randomminzed double blind trial of oral nifedipine and intravenous labetolol in hypertensive emergencies of pregnancy. AJOG.

[14] Svichenko EP, Kupen Hinskala E, Zanozdra NZ, Bezodarak LV, Radchenko W (1992). A comparative analysis of antihypertensive actively of calcium antagonists and adrenoblockers in long term treatment. KLIN Med (MOSK).

[15] Beardmoor KS, Morris JM, Gallery EDM (2002). Excretion of antihypertensive medication into human breast milk;a systematic review. Hypertens Pregnancy.

[16] Hasnai S, Khan M, Saleem A, Waqar M (2009). Prevalence of asthama allergic shinitis among school children of Karachi, Pakistan 2007. J Asthma.

[17] Firoz T, Magee LA, MacDonell K, Payne BA, Gordon R, Vidler M (2014). Oral antihypertensive therapy for severe hypertension in pregnancy and post-partum:a systematic review. BJOG.

[18] Raheem A, Saaid R, Omar S, Tan PC (2012). Oral nifedipine versus intravenous labetolol for acute blood pressure control in hypertensive emergencies of pregnancy:a randomized trial. BJOG.

[19] Cairns AE, Pealing L, Duffy JMN, Roberts N, Tucker KL, Leason P (2017). Post-partum management of hypertensive disorders of pregnancy:a systematic review. BMJS Open.

[20] Vermillion ST, Scardo JA, Newmann RB, Chauhan SP (1999). A randomized double blind trial of oral nifedipine and intravenous labetolol in hypertensive emergencies of pregnancy. Am J Obstet Gynaecol.

[21] Veena P, Perivela L, Raghavan SS (2017). Furosemide in Post-partum management of severe pre-eclampsia:a randomized controlled trial. Hyperten Pregnancy.

[22] Shumard K, Yoon J, Huang C, Nitsche JF (2016). Peripartum antihypertensive choice effects time to blood pressure control in treating hypertensive disorders of pregnancy. Am J Obstet Gynaecol.

[23] Sharma KJ, Greene N, Kilpatrick SJ (2017). Oral Labetolol compared to oral nifedipine for post-partum hypertension. A randomized controlled trial. Hyperten Pregnancy.

[24] Beatty CA, Dangel A (2018). Timely discharge:oral nifedipine is superior to labetolol for post-partum BP control in patients with preeclampsia. Obstet Gynaecol.

